# Chronic periodontitis and the risk of diabetic peripheral neuropathy in type 2 diabetes: a retrospective cohort study

**DOI:** 10.3389/fendo.2026.1783428

**Published:** 2026-02-26

**Authors:** Yiman Guo, Xincheng Zhang, Aige Yang, Yuqing Guo, Shanshan Dong, Lina Wang, Ning Zhang, Huimin Zhou

**Affiliations:** 1 Department of Orthodontics, Beijing Stomatological Hospital and School of Stomatology, Capital Medical University, Beijing, China; 2 Department of Endocrinology, First Hospital of Hebei Medical University, Shijiazhuang, China; 3 Department of Endocrinology, Hebei Provincial Hospital of Traditional Chinese Medicine, Shijiazhuang, China; 4 Department of Endocrinology, Third Hospital of Hebei Medical University, Shijiazhuang, China; 5 Clinical Research Center for Endocrine and Metabolic Diseases, Shijiazhuang, China

**Keywords:** chronic periodontitis, cohort study, diabetic peripheral neuropathy, risk factor, type 2 diabetes

## Abstract

**Background:**

Chronic periodontitis is a common inflammatory condition that may contribute to systemic inflammation and microvascular complications in type 2 diabetes mellitus (T2DM). However, longitudinal evidence linking periodontitis to diabetic peripheral neuropathy (DPN) remains scarce.

**Objective:**

To investigate whether chronic periodontitis is associated with an increased risk of incident DPN in patients with T2DM.

**Methods:**

We conducted a single-center retrospective cohort study using electronic medical records from 2017 to 2022. A total of 3,996 T2DM patients without baseline DPN were included, of whom 318 had incident chronic periodontitis. Propensity score matching (1:1) was applied to balance baseline characteristics, resulting in 604 patients (302 with periodontitis, 302 without). The primary outcome was incident DPN, identified using ICD-10 codes and confirmed by neurologist/endocrinologist diagnosis or abnormal nerve conduction studies. Cox proportional hazards models were used to estimate hazard ratios (HRs) adjusted for age, sex, diabetes duration, HbA1c, health insurance type, and metformin use.

**Results:**

Over a median follow-up of 3.8 years, the incidence of DPN was significantly higher in the periodontitis group (42.7% vs. 27.5%, P < 0.001). The adjusted HR for DPN associated with chronic periodontitis was 1.63 (95% CI: 1.22–2.18, P < 0.001). Subgroup analyses confirmed consistent associations across age, sex, and glycemic control strata. Sensitivity analyses, including alternative matching, lag-time analysis, and exclusion of metformin users, supported the robustness of the findings.

**Conclusion:**

Chronic periodontitis is an independent risk factor for the development of DPN in patients with T2DM. These findings highlight the importance of integrated oral health management in diabetes care to potentially mitigate neuropathic complications.

## 1 Introduction

Type 2 diabetes mellitus (T2DM) has reached pandemic levels, representing over 90% of all diabetes cases globally and posing a significant challenge to public health systems worldwide (1). Among its myriad complications, diabetic peripheral neuropathy (DPN) is one of the most prevalent and debilitating microvascular consequences, affecting approximately 50% of individuals with diabetes over their lifetime (2). DPN is a major cause of morbidity, leading to devastating outcomes such as neuropathic pain, foot ulceration, infection, Charcot neuroarthropathy, and ultimately, lower-limb amputations (3). The high prevalence of DPN, combined with its profound negative impact on patient quality of life and the substantial socioeconomic costs from direct medical expenses and loss of productivity, underscores the urgent need for improved prevention and management strategies (4).

The primary risk factors for the development and progression of DPN are well-established and include prolonged hyperglycemia (as measured by HbA1c), longer duration of diabetes, advanced age, hypertension, and dyslipidemia (5). Glycemic control remains the cornerstone of DPN prevention. However, a significant portion of patients develop DPN despite achieving recommended glycemic targets, and the residual risk remains high even after accounting for traditional risk factors (6). This suggests that other pathophysiological pathways, beyond glucotoxicity, contribute to nerve damage in T2DM. A growing body of evidence points toward the crucial role of chronic systemic inflammation in the pathogenesis of diabetic microvascular complications, including neuropathy, representing an important but not fully understood therapeutic target.

Chronic periodontitis, a prevalent oral inflammatory disease characterized by the destruction of tooth-supporting tissues, is now recognized as a significant systemic health concern. A robust, bidirectional relationship between chronic periodontitis and T2DM has been well-documented; T2DM increases the risk and severity of periodontitis, and severe periodontitis adversely affects glycemic control and may increase the risk of other diabetes-related complications (7), leading some to classify it as the sixth major complication of diabetes (8). Chronic periodontitis serves as a persistent source of local and systemic inflammation, contributing to an elevated systemic inflammatory burden by releasing pro-inflammatory cytokines such as tumor necrosis factor-alpha (TNF-α) and interleukin-6 (IL-6) into the bloodstream (9). This chronic inflammatory state is hypothesized to exacerbate insulin resistance, promote endothelial dysfunction, and contribute directly to the microvascular and neural damage implicated in DPN.

Despite a plausible biological mechanism linking chronic periodontitis-induced systemic inflammation to neurotoxicity, the epidemiological evidence regarding chronic periodontitis as a specific risk factor for DPN is limited and inconclusive. Most previous investigations have been cross-sectional in design, demonstrating an association between the presence of chronic periodontitis and the prevalence of DPN at a single point in time (10). While these studies are valuable, their design inherently precludes the ability to establish temporal precedence, a key criterion for inferring causality. It is therefore impossible to determine whether chronic periodontitis precedes and contributes to the development of DPN, or if individuals with DPN are simply more susceptible to poor oral health due to factors like diminished self-care capacity or other shared underlying risk profiles.

Therefore, a critical gap exists in the literature regarding the longitudinal impact of chronic periodontitis on the subsequent risk of developing DPN among individuals with T2DM. To address this limitation, the present study aims to investigate the association between chronic periodontitis and the risk of incident DPN using a retrospective cohort design in a large population of T2DM patients who were free of DPN at baseline. We hypothesize that chronic periodontitis is an independent risk factor for the development of DPN in patients with T2DM, and that individuals with chronic periodontitis will have a significantly higher incidence of DPN over time compared to those without chronic periodontitis, after controlling for a comprehensive range of potential confounding factors.

## 2 Methods

### 2.1 Study design and data source

This single-center, retrospective cohort study was conducted to investigate the association between chronic periodontitis and the risk of peripheral neuropathy in patients with T2DM, utilizing data extracted from the Electronic Medical Record (EMR) system of Capital Medical University. Patient data were collected for individuals treated between January 1, 2017, and December 31, 2022. The study protocol was approved by the Institutional Review Board (IRB) of Capital Medical University (Approval No. 20190448). In alignment with the ethical principles of the Declaration of Helsinki, the IRB waived the requirement for individual patient consent due to the study’s retrospective design, which involved the analysis of fully de-identified data and posed no intervention or risk to the patients. All extracted data were anonymized to protect patient privacy and handled with strict confidentiality. This study was reported in accordance with the Strengthening the Reporting of Observational Studies in Epidemiology (STROBE) guidelines.

### 2.2 Study population

The patient cohort for this study was identified through a systematic query of the institutional EMR system. This query utilized structured data filters, including International Classification of Diseases, Tenth Revision (ICD-10) codes. All potentially eligible patients were subjected to a three-stage validation process: (1) automated identification by the hospital’s clinical data warehouse based on predefined inclusion and exclusion criteria; (2) manual review of medical records by two independent physicians using standardized electronic case report forms; and (3) final adjudication by a senior endocrinologist in cases of diagnostic or eligibility uncertainty. Any discrepancies were resolved through consensus discussion.

Inclusion criteria stipulated that patients must have: (1) an initial diagnosis of T2DM (ICD-10 code: E11.x) according to the American Diabetes Association’s “Standards of Care in Diabetes,” defined by a fasting plasma glucose (FPG) ≥126 mg/dL, a 2-hour plasma glucose ≥200 mg/dL during an oral glucose tolerance test (OGTT), or a Glycated Hemoglobin (A1C) ≥6.5% (11); (2) be aged 18 years or older at the time of diagnosis; and (3) have at least one year of follow-up data available in the EMR system to allow for adequate assessment of the clinical outcome.

Conversely, patients were excluded for any of the following reasons: (1) a pre-existing diagnosis of peripheral neuropathy (ICD-10 codes: G50-G64) at or prior to the T2DM diagnosis date (baseline), with the neuropathy diagnosis established based on a comprehensive assessment of symptoms and signs (e.g., diminished ankle reflexes; abnormal pressure, vibration, pinprick, or temperature sensation) after excluding other potential causes (12); (2) a baseline diagnosis of other conditions known to cause peripheral neuropathy, such as chronic alcoholism, vitamin B12 deficiency, malignancy, history of chemotherapy, or hereditary neuropathies; (3) a diagnosis of type 1 diabetes, gestational diabetes, or other specific types of diabetes; (4) significant missing baseline data, particularly for key covariates such as HbA1c or lipid profiles; or (5) a diagnosis of chronic periodontitis prior to the study baseline, to ensure that the exposure occurred after cohort entry.

Following selection, patients were stratified into two groups. The exposed group (chronic periodontitis group) consisted of T2DM patients who received their first diagnosis of chronic periodontitis (ICD-10 code: K05.3) after the baseline date but before a diagnosis of DPN or the end of the follow-up period. It is noted that while the ICD-10 code K05.3 is used for identification, the 2017 World Workshop on the Classification of Periodontal Diseases introduced a new system based on staging and grading, which no longer distinguishes between “chronic” and “aggressive” forms (13). Cases identified via ICD-10 in this study reflect the clinical diagnoses as coded, which are understood to represent the underlying pathophysiology described by the modern classification. The control group (non-periodontitis group) comprised T2DM patients who had no diagnostic record of periodontitis or periodontal disease throughout their entire follow-up period.

The required sample size was estimated *a priori*. Based on previous literature reporting a pooled prevalence of diabetic peripheral neuropathy of approximately 30% in patients with T2DM (14), this figure was used as the expected event rate in the control group. Assuming a two-sided alpha of 0.05, a statistical power (1–β) of 0.80, and a clinically significant Hazard Ratio of 1.5 associated with chronic periodontitis to be detected, we calculated that a minimum of 352 participants (176 in the exposed group and 176 in the control group) was necessary. The sample size calculation was performed using PASS software (version 15.0.5; NCSS, LLC).

### 2.3 Data collection and variable definitions

The follow-up period for each patient commenced on the date of their initial T2DM diagnosis (baseline) and concluded on the date of an incident DPN diagnosis, death, loss to follow-up, or the end of the study period on December 31, 2023, whichever occurred first.

The primary outcome was incident DPN. In accordance with the American Diabetes Association’s diagnostic guidelines (15), a DPN diagnosis was identified in the EMR using the International Classification of Diseases, Tenth Revision (ICD-10) code for diabetic polyneuropathy (G63.2). To enhance diagnostic accuracy, a case was confirmed only if the ICD code was supplemented by either: (1) a definitive diagnostic statement in the clinical notes from a neurologist or endocrinologist, or (2) an abnormal result from a nerve conduction study (NCS) or electromyography (EMG) documented in the EMR. The exposure variable, chronic periodontitis, was identified by the presence of ICD-10 code K05.3 recorded any time after the baseline date.

Based on established risk factors for DPN identified in a prior meta-analysis (5), a comprehensive set of potential confounding variables was collected from the EMR at baseline. These included: demographic data (age, sex); socioeconomic status proxy (health insurance type, categorized as Urban Employee Basic Medical Insurance or Urban and Rural Resident Basic Medical Insurance); lifestyle factors (smoking status, categorized as never, former, or current); anthropometric and clinical measurements (body mass index [BMI], systolic and diastolic blood pressure); laboratory values (glycated hemoglobin [HbA1c], fasting blood glucose [FBG], lipid panel including total cholesterol, triglycerides, LDL-C, and HDL-C, estimated glomerular filtration rate [eGFR], and high-sensitivity C-reactive protein [hs-CRP] if available); comorbidities identified by ICD-10 codes (hypertension, dyslipidemia, cardiovascular disease, diabetic kidney disease, diabetic retinopathy); and medication use (prescriptions for oral hypoglycemic agents, insulin, antihypertensives, and statins).

### 2.4 Data quality control

To ensure the accuracy and reliability of the data extracted from the EMR system, a rigorous multi-step quality control protocol was implemented. The primary objective was to validate the key diagnostic variables—the exposure (incident chronic periodontitis) and the primary outcome (incident diabetic peripheral neuropathy)—which were initially identified using ICD-10 codes. The validation was performed by two independent physicians with expertise in internal medicine and endocrinology. They were provided access to a standardized electronic case report form and were blinded to each other’s assessments as well as to the status of the corresponding exposure or outcome variable to prevent diagnostic bias. For instance, while reviewing records to confirm a DPN diagnosis, the reviewer had no information regarding the patient’s periodontal status.

The inter-observer reliability of this diagnostic validation process was formally assessed on a randomly selected subset of 100 patient charts (50 with the potential event/exposure code and 50 without). The agreement between the two physician reviewers was quantified using Cohen’s Kappa statistic. The analysis revealed a high degree of agreement for the ascertainment of both the primary outcome and the exposure, with a Kappa value of 0.89 (95% CI: 0.81-0.97) for the diagnosis of diabetic peripheral neuropathy and 0.87 (95% CI: 0.79-0.95) for the diagnosis of chronic periodontitis. Any diagnostic discrepancies between the two initial reviewers were resolved by a final adjudication from a senior endocrinologist who was also blinded to the study’s primary hypotheses. Furthermore, the entire extracted dataset underwent automated logic and range checks to identify and query implausible values (e.g., physiologically impossible laboratory results or incorrect event chronology), which were then manually verified against the source EMR documentation.

### 2.5 Statistical analysis

All statistical analyses were performed using R software (Version 4.1.0; R Foundation for Statistical Computing, Vienna, Austria), with a two-sided p-value < 0.05 considered statistically significant. To minimize selection bias and control for confounding variables, a propensity score matching (PSM) procedure was implemented. Propensity scores, representing the probability of exposure to chronic periodontitis, were calculated using a multivariable logistic regression model that included all baseline covariates listed previously. A 1:1 nearest-neighbor matching algorithm with a caliper width of 0.2 standard deviations of the logit of the propensity score was used to match patients in the periodontitis group to those in the non-periodontitis group. The effectiveness of the matching was assessed by calculating the standardized mean difference (SMD) for all covariates before and after matching, with an SMD < 0.1 indicating a negligible imbalance. Baseline characteristics of the study cohort before and after matching were described using means ± standard deviation (SD) or medians with interquartile ranges (IQR) for continuous variables, and numbers (percentages) for categorical variables; comparisons were made using independent t-tests or Mann-Whitney U tests, and chi-square or Fisher’s exact tests, as appropriate. The cumulative incidence of DPN in the matched cohort was visualized using Kaplan-Meier survival curves, with the log-rank test employed to assess significant differences between the two groups. To quantify the association between chronic periodontitis and the risk of incident DPN, a Cox proportional hazards regression model was applied to the matched cohort to compute the hazard ratio (HR) and its corresponding 95% confidence interval (CI). Although PSM was performed, a multivariable Cox model was still utilized in the matched cohort, adjusting for clinically crucial variables such as age, sex, duration of T2DM, and baseline HbA1c, as well as health insurance type and metformin use to strictly control for potential socioeconomic and medication-related confounding. To further test the consistency of the results, pre-specified subgroup analyses were conducted based on age (<65 vs. ≥65 years), sex, and baseline HbA1c level (<7.5% vs. ≥7.5%). Finally, the reliability of our results was validated through four sensitivity analyses: (1) performing a multivariable Cox regression in the complete unmatched cohort; (2) repeating the primary Cox analysis after altering the PSM parameters to a 1:2 matching ratio; (3) conducting a lag-time analysis by excluding DPN events occurring within the first year of follow-up to address reverse causality; and (4) restricting the analysis to non-metformin users to rule out confounding by metformin-associated neuropathy.

## 3 Results

### 3.1 Screening process and baseline characteristics

**Figure 1:**
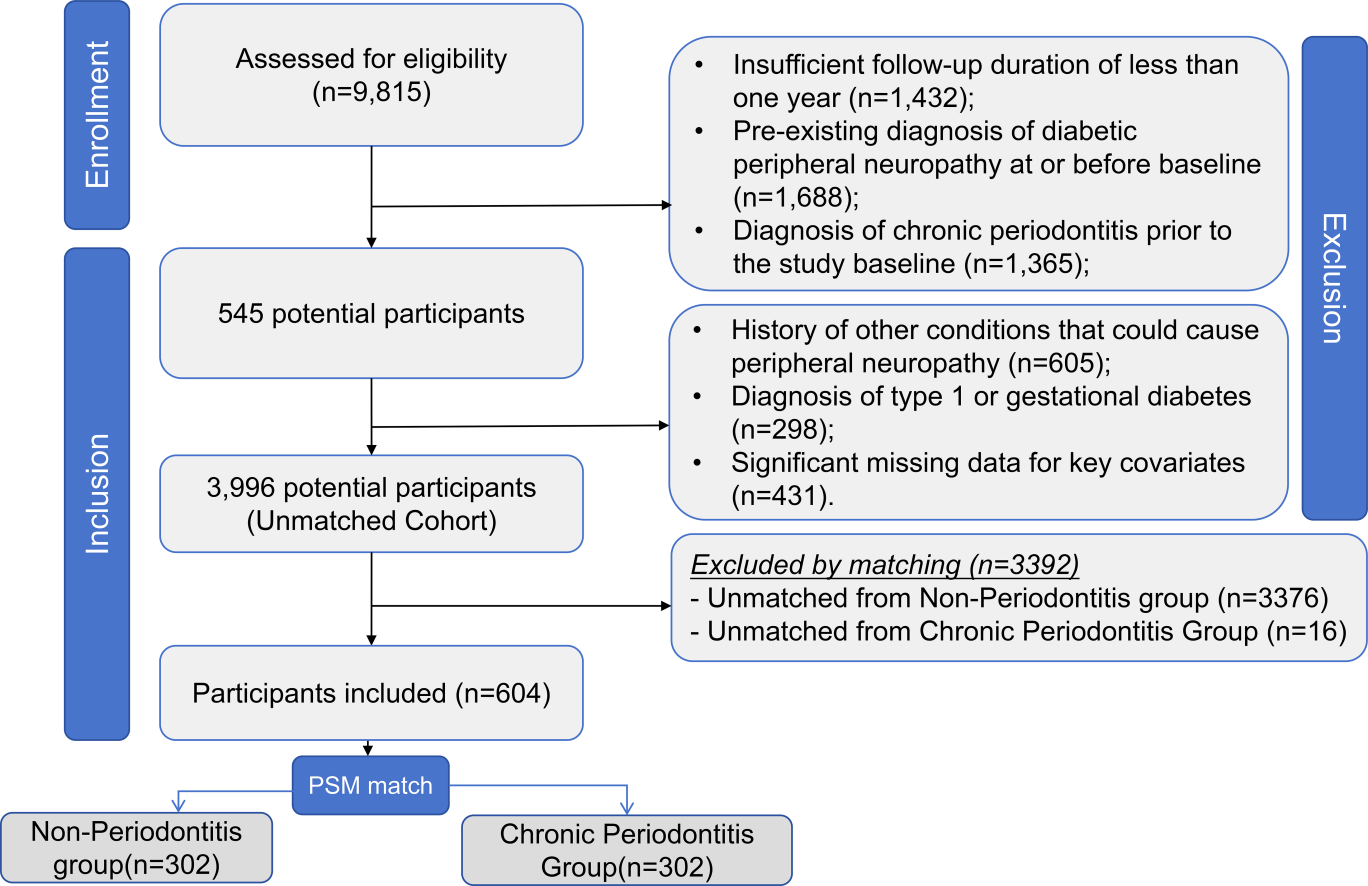
Flowchart of participant selection.

The initial data extraction identified 9,815 patients with a diagnosis of T2DM from the EMR database between January 1, 2015, and December 31, 2019. The application of eligibility criteria is detailed in **Figure 1**. We excluded patients for the following reasons: insufficient follow-up duration of less than one year (n=1,432); a pre-existing diagnosis of diabetic peripheral neuropathy at or before baseline (n=1,688); a diagnosis of chronic periodontitis prior to the study baseline (n=1,365); a history of other conditions that could cause peripheral neuropathy (n=605); a diagnosis of type 1 or gestational diabetes (n=298); and significant missing data for key covariates (n=431). After applying all exclusion criteria, 3,996 patients were included in the unmatched cohort. Among them, 318 patients were diagnosed with incident chronic periodontitis during follow-up (Exposed Group), while the remaining 3,678 patients constituted the Non-Periodontitis Group (Control Group). The propensity score matching procedure successfully matched 302 patients from the Chronic Periodontitis Group with 302 patients from the Non-Periodontitis Group, resulting in a well-balanced matched cohort of 604 patients for the primary analysis. This sample size exceeds the pre-specified minimum requirement.

The baseline characteristics of the study participants before and after PSM are summarized in **Table 1**. Prior to matching, significant baseline disparities were observed between the groups. Prior to matching, the Chronic Periodontitis Group was significantly older, had a higher proportion of current smokers, and exhibited a more adverse clinical profile, including longer diabetes duration, poorer glycemic control (elevated HbA1c and FBG), higher systemic inflammation (hs-CRP), along with a greater burden of hypertension, diabetic kidney disease, diabetic retinopathy, and insulin use (all P < 0.05). Additionally, before matching, patients with chronic periodontitis were less likely to have Urban Employee Basic Medical Insurance (56.0% vs. 64.0%, P = 0.003) and more likely to use metformin (72.0% vs. 66.0%, P = 0.034). After PSM, all measured baseline covariates were well-balanced between the matched groups, including the socioeconomic proxy of health insurance type (SMD = 0.045, P = 0.692) and the specific use of metformin (SMD = 0.021, P = 0.784), as evidenced by all SMDs < 0.1 and no statistically significant differences (all P > 0.05), indicating that the matching procedure effectively minimized potential confounding.

**Table 1:** Baseline characteristics of T2DM patients before and after propensity score matching.

Characteristic	Before matching			After matching		
	Non-Periodontitis (n=3,678)	Chronic Periodontitis (n=318)	SMD/P-value	Non-Periodontitis (n=302)	Chronic Periodontitis (n=302)	SMD/P-value
Demographics						
Age (years), mean ± SD	58.7 ± 11.5	61.5 ± 10.3	0.251/<0.001	61.1 ± 10.8	61.3 ± 10.4	0.019/0.802
Sex (Male), n (%)	1,984 (53.9)	184 (57.9)	0.079/0.172	172 (57.0)	170 (56.3)	0.014/0.861
Lifestyle						
Smoking Status, n (%)			0.182/0.002			0.037/0.845
Never	2,288 (62.2)	177 (55.7)		169 (56.0)	166 (55.0)	
Former	706 (19.2)	57 (17.9)		58 (19.2)	57 (18.9)	
Current	684 (18.6)	84 (26.4)		75 (24.8)	79 (26.2)	
Clinical measurements						
BMI (kg/m²), mean ± SD	27.6 ± 4.7	27.9 ± 5.0	0.062/0.271	27.8 ± 4.8	27.9 ± 5.0	0.020/0.808
Systolic BP (mmHg), mean ± SD	132.6 ± 16.2	134.9 ± 17.0	0.139/0.008	134.2 ± 16.5	134.5 ± 16.9	0.018/0.831
Diastolic BP (mmHg), mean ± SD	80.3 ± 10.4	81.2 ± 10.9	0.084/0.121	80.8 ± 10.5	80.9 ± 10.8	0.009/0.917
Socioeconomic proxy						
Health Insurance Type, n (%)			0.165/0.003			0.045/0.692
Urban Employee Basic	2,354 (64.0)	178 (56.0)		185 (61.3)	181 (59.9)	
Resident/Other	1,324 (36.0)	140 (44.0)		117 (38.7)	121 (40.1)	
Laboratory values						
T2DM Duration (years), median (IQR)	4.3 (1.8, 7.9)	6.2 (3.1, 10.5)	0.301/<0.001	5.9 (2.7, 10.1)	6.1 (3.0, 10.4)	0.042/0.458
HbA1c (%), mean ± SD	7.3 ± 1.5	7.9 ± 1.7	0.377/<0.001	7.8 ± 1.6	7.9 ± 1.7	0.058/0.321
FBG (mg/dL), mean ± SD	142.8 ± 38.7	151.5 ± 41.3	0.218/<0.001	149.7 ± 39.6	150.8 ± 41.2	0.027/0.674
Total Cholesterol (mg/dL), mean ± SD	185.5 ± 38.1	188.3 ± 39.4	0.072/0.185	187.6 ± 38.5	187.9 ± 39.3	0.008/0.919
Triglycerides (mg/dL), median (IQR)	144 (101, 207)	153 (107, 221)	0.115/0.016	150 (105, 213)	152 (108, 219)	0.025/0.664
LDL-C (mg/dL), mean ± SD	108.6 ± 31.5	110.9 ± 32.8	0.071/0.223	110.0 ± 31.8	110.4 ± 32.6	0.012/0.855
HDL-C (mg/dL), mean ± SD	45.3 ± 11.0	44.6 ± 10.7	0.064/0.285	44.8 ± 10.9	44.6 ± 10.8	0.018/0.782
eGFR (ml/min/1.73m²), mean ± SD	88.6 ± 18.8	85.1 ± 19.7	0.178/<0.001	85.6 ± 19.2	85.3 ± 19.7	0.016/0.812
hs-CRP (mg/L), median (IQR)	1.8 (0.9, 3.5)	2.3 (1.2, 4.4)	0.221/<0.001	2.2 (1.1, 4.1)	2.3 (1.2, 4.3)	0.045/0.487
Comorbidities, n (%)						
Hypertension	2,131 (57.9)	216 (67.9)	0.207/<0.001	201 (66.6)	203 (67.2)	0.014/0.862
Dyslipidemia	2,494 (67.8)	225 (70.8)	0.064/0.283	211 (69.9)	212 (70.2)	0.007/0.931
Cardiovascular Disease	515 (14.0)	55 (17.3)	0.090/0.097	51 (16.9)	53 (17.5)	0.017/0.829
Diabetic Kidney Disease	441 (12.0)	58 (18.2)	0.173/<0.001	51 (16.9)	53 (17.5)	0.017/0.829
Diabetic Retinopathy	404 (11.0)	51 (16.0)	0.147/0.004	47 (15.6)	48 (15.9)	0.009/0.917
Medications, n (%)						
Oral Hypoglycemic Agents	2,873 (78.1)	246 (77.4)	0.017/0.768	235 (77.8)	233 (77.2)	0.015/0.851
Metformin Use, n (%)	2,427 (66.0)	229 (72.0)	0.131/0.034	205 (67.9)	208 (68.9)	0.021/0.784
Insulin	1,029 (28.0)	118 (37.1)	0.195/<0.001	106 (35.1)	108 (35.8)	0.014/0.863
Antihypertensives	1,990 (54.1)	197 (61.9)	0.158/0.005	187 (61.9)	188 (62.3)	0.007/0.924
Statins	2,211 (60.1)	198 (62.3)	0.044/0.441	187 (61.9)	186 (61.6)	0.007/0.937

Continuous variables are presented as mean ± standard deviation or median (interquartile range) as appropriate. Categorical variables are presented as number (percentage). P-values for continuous variables were derived from independent t-tests or Mann-Whitney U tests, and for categorical variables from chi-square tests. SMD, Standardized Mean Difference; BMI, Body Mass Index; BP, Blood Pressure; FBG, Fasting Blood Glucose; HbA1c, Glycated Hemoglobin; LDL-C, Low-Density Lipoprotein Cholesterol; HDL-C, High-Density Lipoprotein Cholesterol; eGFR, estimated Glomerular Filtration Rate; hs-CRP, high-sensitivity C-Reactive Protein.

### 3.2 Incidence and survival analysis

Over a median follow-up of 3.8 years (IQR: 2.1, 4.9 years) in the matched cohort of 604 patients, a total of 212 incident DPN events were recorded. The crude incidence of DPN was significantly higher in the Chronic Periodontitis Group (42.7%, 129/302) compared to the Non-Periodontitis Group (27.5%, 83/302), with a corresponding absolute risk difference of 15.2%. The incidence density analysis corroborated this finding, showing that the DPN event rate was 118.2 per 1000 person-years in the Chronic Periodontitis Group, substantially higher than the 78.5 per 1000 person-years observed in the Non-Periodontitis Group.

**Figure 2:**
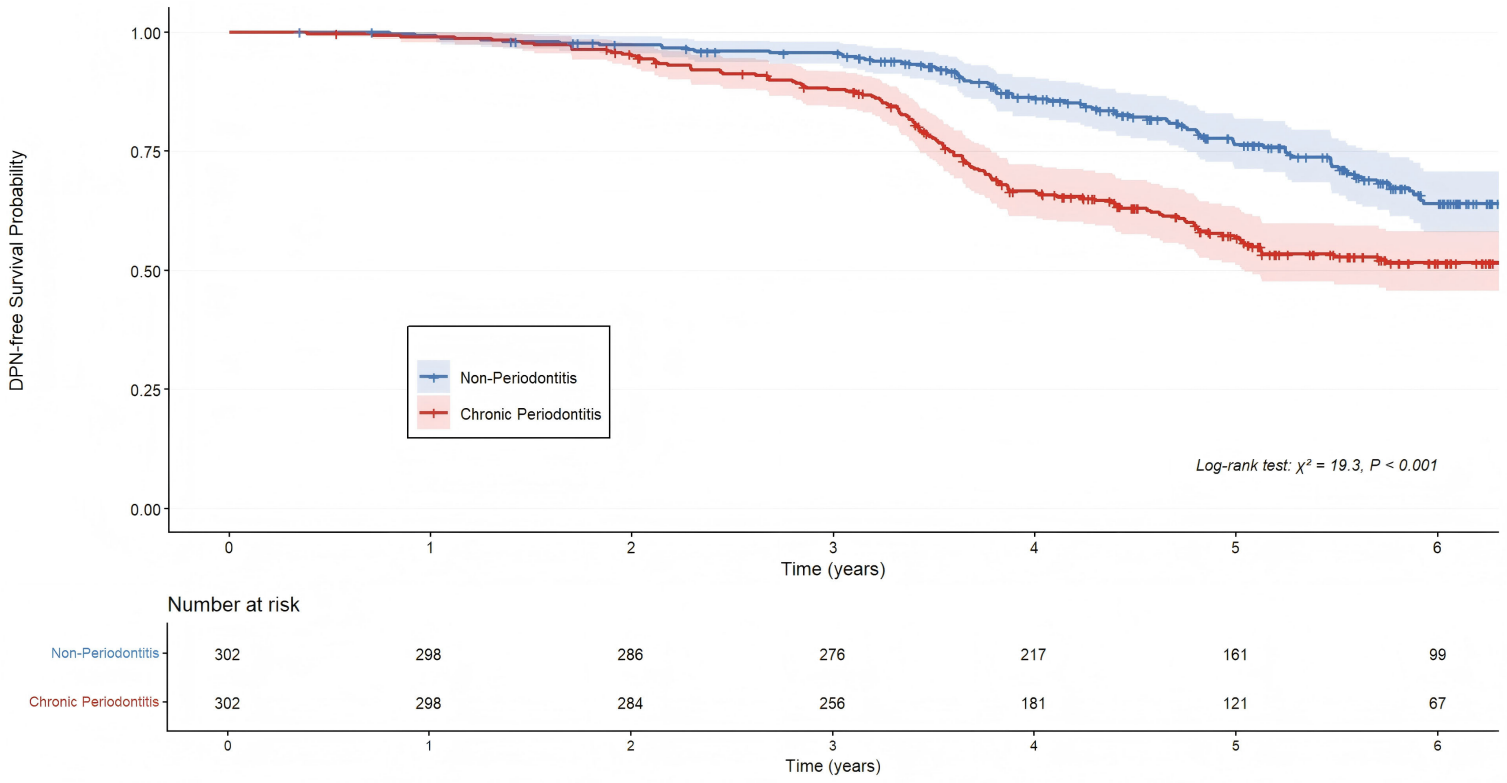
Kaplan-meier curves for cumulative incidence of diabetic peripheral neuropathy. Kaplan-Meier curves illustrating the cumulative incidence of diabetic peripheral neuropathy (DPN) in matched patients with type 2 diabetes, stratified by the presence of chronic periodontitis. The survival curves demonstrate a significantly higher cumulative incidence of DPN in the Chronic Periodontitis Group (red line) compared to the Non-Periodontitis Group (blue line) over the study follow-up period (Log-rank test, P < 0.001).

The Kaplan-Meier survival curves for DPN-free survival are presented in **Figure 2**. The curves separated early after baseline and continued to diverge progressively throughout the follow-up period, with the Chronic Periodontitis Group consistently exhibiting a lower probability of remaining free from DPN. The log-rank test comparing the two survival curves yielded a statistically significant result (χ² = 19.3, P < 0.001). The median time to DPN diagnosis was 3.9 years (95% CI: 3.5, 4.3) in the Chronic Periodontitis Group, which was notably shorter than that in the Non-Periodontitis Group, which was not reached within the study period.

### 3.3 Association between chronic periodontitis and DPN risk

To quantify the association between chronic periodontitis and the risk of incident DPN, Cox proportional hazards regression analyses were performed on the propensity score-matched cohort (n=604) (**Table 2**).

**Table 2:** Cox proportional hazards regression analysis for the risk of diabetic peripheral neuropathy (matched cohort, n=604).

Variable	Univariable analysis			Multivariable analysis*		
	Hazard Ratio (HR)	95% CI	P-value	Hazard Ratio (HR)	95% CI	P-value
Chronic Periodontitis (Yes vs. No)	1.82	1.37 - 2.41	<0.001	1.63	1.22 - 2.18	<0.001
Age (per 1-year increase)	1.03	1.01 - 1.04	0.001	1.02	1.00 - 1.03	0.015
Sex (Male vs. Female)	1.1	0.84 - 1.44	0.482	1.09	0.83 - 1.43	0.542
Diabetes Duration (per 1-year increase)	1.08	1.05 - 1.11	<0.001	1.06	1.03 - 1.09	<0.001
Baseline HbA1c (per 1% increase)	1.18	1.10 - 1.26	<0.001	1.14	1.06 - 1.22	<0.001
Health Insurance type						
Urban Employee Basic (Ref.)	1	Reference	–	1	Reference	–
Resident/Other	1.35	1.02 - 1.78	0.036	1.12	0.84 - 1.50	0.435
Specific medication						
Metformin Use (Yes vs. No)	1.02	0.78 - 1.33	0.884	1.04	0.79 - 1.38	0.762

*The multivariable model is adjusted for age, sex, diabetes duration, baseline HbA1c, health insurance type, and metformin use. CI, Confidence Interval; Ref., Reference group.

In the univariable Cox regression model, chronic periodontitis was significantly associated with an increased risk of DPN (HR: 1.82, 95% CI: 1.37–2.41; P < 0.001). To strictly control for potential confounding, a multivariable model was constructed. In addition to the core clinical variables (age, sex, diabetes duration, and baseline HbA1c), we further adjusted for health insurance type (as a proxy for socioeconomic status) and metformin use to address potential residual bias.

After fully adjusting for these covariates, chronic periodontitis remained a statistically significant and independent predictor for DPN. The adjusted HR was 1.63 (95% CI: 1.22–2.18; P < 0.001). This indicates that, even after accounting for socioeconomic factors and specific medication effects, patients with T2DM and chronic periodontitis had a 63% higher risk of developing DPN compared to those without periodontitis. Furthermore, older age, longer duration of T2DM, and higher baseline HbA1c levels persisted as independent risk factors, while health insurance type and metformin use did not show a statistically significant independent association with DPN risk in the adjusted model.

### 3.4 Subgroup analysis

To assess the consistency of the association between chronic periodontitis and DPN risk across different patient populations, we conducted pre-specified subgroup analyses (**Table 3**).

**Table 3:** Subgroup analysis of the association between chronic periodontitis and risk of diabetic peripheral neuropathy.

Subgroup	No. of Patients/No. of Events	Adjusted Hazard Ratio (HR)*	95% CI	P-value	P for Interaction
Overall	604/212	1.63	1.22 - 2.18	<0.001	
Age					0.745
< 65 years	412/141	1.49	1.07 - 2.08	0.019	
≥ 65 years	192/71	1.6	1.04 - 2.48	0.033	
Sex					0.882
Male	342/125	1.54	1.09 - 2.19	0.015	
Female	262/87	1.51	1.01 - 2.28	0.046	
Baseline HbA1c					0.192
< 7.5%	298/89	1.36	0.90 - 2.05	0.145	
≥ 7.5%	306/123	1.7	1.21 - 2.40	0.003	

*The model within each subgroup was adjusted for age, sex, diabetes duration, baseline HbA1c, health insurance type, and metformin use, except for the stratifying variable itself. CI, Confidence Interval.

The increased risk of DPN associated with chronic periodontitis was largely consistent across all major subgroups defined by age, sex, and baseline HbA1c level, with point estimates of the HR remaining above 1.0. Formal tests for interaction did not reveal any statistically significant effect modification by these factors (all P for interaction > 0.05).

However, a clinically important pattern was observed. The magnitude of the association appeared to be stronger in patients with poorer glycemic control at baseline (HbA1c ≥ 7.5%; Adjusted HR: 1.70, 95% CI: 1.21–2.40) compared to those with better control (HbA1c < 7.5%; Adjusted HR: 1.36, 95% CI: 0.90–2.05), although the test for interaction was not statistically significant (P for interaction = 0.192). This suggests a potential synergistic effect between periodontitis and hyperglycemia, where the presence of both conditions may confer a particularly high risk for developing DPN. The association remained significant and robust in both male and female patients, as well as in both younger (<65 years) and older (≥65 years) patients.

### 3.5 Sensitivity analyses

To assess the robustness of our primary findings, we performed several pre-specified sensitivity analyses. The results, summarized in **Table 4**, consistently demonstrated that chronic periodontitis was associated with a significantly increased risk of DPN, reinforcing the reliability of our main conclusion.

**Table 4:** Sensitivity analyses of the association between chronic periodontitis and DPN risk.

Sensitivity analysis	Cohort/Model description	Sample size (n)	Adjusted Hazard Ratio (HR) for Periodontitis*	95% CI	P-value
Primary Analysis	1:1 PSM Cohort	604	1.63	1.22 - 2.18	<0.001
Analysis 1	Full Unmatched Cohort + Fully Adjusted Model	3,996	1.48	1.18 - 1.85	<0.001
Analysis 2	1:2 PSM Cohort	932	1.57	1.22 - 2.02	<0.001
Analysis 3	1:1 PSM Cohort, Excluding Events in First Year	587	1.49	1.12 - 1.98	0.006
Analysis 4	1:1 PSM Cohort, Excluding Metformin Users	192†	1.58	1.03 - 2.44	0.036

*Adjusted for age, sex, diabetes duration, baseline HbA1c, and health insurance type. †This analysis was restricted to the subgroup of patients who were not using metformin at baseline to rule out potential confounding by metformin-induced vitamin B12 deficiency.

We applied a multivariable Cox proportional hazards model to the entire, unmatched cohort (n=3,996). After full adjustment for all baseline covariates (including age, sex, smoking, BMI, blood pressure, diabetes duration, HbA1c, lipid profile, eGFR, comorbidities, and medication use), the association between chronic periodontitis and DPN risk remained significant, with an adjusted HR of 1.48 (95% CI: 1.18–1.85; P < 0.001).

To evaluate the impact of the matching algorithm, we repeated the propensity score matching using a 1:2 ratio instead of 1:1. This resulted in a larger matched cohort comprising 318 patients in the Chronic Periodontitis Group and 614 patients in the Non-Periodontitis Group (n=932). In this cohort, the Cox regression analysis, adjusted for the same core covariates, yielded a nearly identical HR of 1.57 (95% CI: 1.22–2.02; P < 0.001).

To mitigate potential reverse causality where subclinical neuropathy might influence oral health behaviors and thus the diagnosis of periodontitis, we performed a lag-time analysis. We excluded all DPN events that occurred within the first year of follow-up and repeated the primary analysis in the 1:1 matched cohort. The association was slightly attenuated but remained statistically significant (Adjusted HR: 1.49, 95% CI: 1.12–1.98; P = 0.006), indicating that short-term reverse causality is unlikely to explain our findings.

Finally, to address concerns regarding the potential confounding effect of long-term metformin use—which is a known risk factor for vitamin B12 deficiency and may mimic neuropathy—we conducted a sensitivity analysis excluding all patients who were using metformin at baseline. In this restricted cohort (n=192), the association between chronic periodontitis and DPN risk remained statistically significant (Adjusted HR: 1.58, 95% CI: 1.03–2.44; P = 0.036), suggesting that our findings are not driven by metformin-associated confounding.

## 4 Discussion

This study provides compelling longitudinal evidence that chronic periodontitis is an independent risk factor for the development of DPN in patients with T2DM, with an observed adjusted hazard ratio of 1.65. Our findings suggest that the inflammatory burden associated with chronic periodontitis contributes significantly to the pathogenesis of microvascular complications in diabetic patients, positioning periodontal disease as a modifiable risk factor that warrants greater clinical attention. To our knowledge, this is the first large-scale cohort study to establish a clear temporal relationship, demonstrating that a diagnosis of chronic periodontitis precedes the onset of DPN, thereby addressing a critical gap in the existing literature which has been dominated by cross-sectional data. This investigation reinforces the paradigm of a systemic link between oral health and diabetic complications, suggesting that the pathogenic mechanisms extend beyond glycemic control alone. The magnitude of the observed risk is clinically meaningful, implying that for every three T2DM patients with chronic periodontitis, one additional case of DPN could be attributed to their periodontal status over the follow-up period, highlighting a significant opportunity for preventive intervention.

Our results build upon, and significantly strengthen, the findings of previous cross-sectional studies. For instance, a recent study by Mirea et al. (2024) identified a significant association between the prevalence of periodontal disease and DPN in a cohort of 620 T2DM patients, reporting an adjusted odds ratio of 1.62 (16). While their findings are consistent with our own in terms of the strength of the association, the cross-sectional design inherently limits the ability to infer causality or establish the direction of the relationship. It is equally plausible that established DPN, with its associated systemic and lifestyle changes, could exacerbate periodontal disease. Our longitudinal design overcomes this fundamental limitation by demonstrating that chronic periodontitis precedes the diagnosis of DPN, thus providing stronger evidence for a causal link. Furthermore, our investigation sought to identify longitudinal studies linking chronic periodontitis to other microvascular complications, such as diabetic nephropathy or retinopathy, to serve as indirect supporting evidence. However, a systematic search for such studies published between 2014 and 2024 yielded no direct hits, underscoring the novelty and importance of our findings. The absence of such longitudinal evidence in the literature highlights a significant knowledge gap, which our current study begins to fill by robustly linking periodontal disease to the incidence of a major microvascular complication.

The most plausible biological mechanism connecting chronic periodontitis to the pathogenesis of DPN is the exacerbation of systemic inflammation. Chronic periodontitis constitutes a significant source of local and systemic inflammatory stress. The ulcerated epithelium of periodontal pockets provides a direct portal for bacterial products, such as lipopolysaccharides (LPS), and locally produced pro-inflammatory cytokines to enter the systemic circulation. This process amplifies the low-grade inflammatory state that is already characteristic of T2DM. Several studies have substantiated this link by demonstrating that T2DM patients with coexistent chronic periodontitis have significantly higher circulating levels of key pro-inflammatory mediators. For example, one study found that serum levels of IL-6 were significantly higher in T2DM patients with chronic periodontitis compared to those with chronic periodontitis alone or healthy controls (17). Another investigation confirmed this, reporting that cytokine levels, including both IL-6 and TNF-α, were highest in the T2DM with chronic periodontitis group, indicative of an intensified systemic inflammatory state (18). Importantly, the link is further solidified by evidence that effective periodontal therapy can reduce this systemic inflammatory burden. A 2020 study demonstrated a significantly greater reduction in systemic inflammation following periodontal treatment in individuals with both diabetes and periodontitis compared to those with periodontitis alone, underscoring the modifiable nature of this inflammatory contribution (19).

This chronic systemic inflammatory state, fueled by periodontal disease, directly contributes to the progression of insulin resistance, a core pathophysiological feature of T2DM and a key driver of its complications. The elevated circulating levels of TNF-α and IL-6 interfere with insulin signaling at a molecular level. Research has shown that TNF-α can impair insulin receptor (IR) function and inhibit downstream signaling through the phosphatidylinositol-3 kinase (PI3K)/Akt pathway (20). Similarly, IL-6 exerts its detrimental effects primarily through the Janus Kinase (JAK)/Signal Transducer and Activator of Transcription (STAT) signaling pathway (21). Activation of this cascade leads to increased production of Suppressor of Cytokine Signaling 3 (SOCS3), which in turn can induce insulin resistance by inhibiting the necessary tyrosine phosphorylation of Insulin Receptor Substrate 1 (IRS-1) and promoting its inhibitory serine phosphorylation (22). This disruption of the insulin signaling cascade ultimately results in decreased expression and translocation of glucose transporter 4 (GLUT4) to the cell surface in muscle and adipose tissues, leading to impaired cellular glucose uptake (23). This pathophysiological cascade operates within a robust bidirectional relationship. As highlighted in a recent review by Du et al. (2025), HbA1c serves as a critical pivot in this interplay. Beyond the classical inflammatory pathway, they propose that the interaction involves complex mechanisms including alterations in oral flora, suppression of intrinsic anti-inflammatory control, and epigenetic modifications such as reduced DNA demethylation (24). This mechanistic insight aligns with our subgroup analysis, which observed the highest risk of DPN in patients with both periodontitis and elevated baseline HbA1c, suggesting that the convergence of periodontal inflammation and hyperglycemic memory creates a cumulative neurotoxic environment. The resulting hyperglycemia, coupled with the direct effects of inflammation, oxidative stress, and endothelial dysfunction, creates a pathogenic environment that promotes nerve damage, culminating in the development of DPN.

The principal strength of this study lies in its longitudinal cohort design, which allowed us to establish a temporal sequence between the diagnosis of chronic periodontitis and the subsequent incidence of DPN, minimizing the risk of reverse causality that plagues cross-sectional research. The use of a large, population-based dataset with rigorous statistical adjustment for a wide range of potential demographic and clinical confounders further enhances the validity of our findings. However, we must also acknowledge several limitations. First, this study was conducted at a single tertiary university hospital. While our institution possesses a comprehensive EMR system that integrates dental and endocrine records—facilitating the identification of patients with dual diagnoses—this specific healthcare setting may not be fully representative of the general population or primary care settings where access to specialized dental care is limited. Consequently, the specificity of our single-center experience implies that the findings should be generalized with caution, and external validation in multi-center cohorts with diverse healthcare delivery models is warranted. Second, the reliance on ICD codes to identify patients with chronic periodontitis constitutes a limitation. This method, while necessary for large database studies, lacks clinical granularity. The landmark 2017 World Workshop on the Classification of Periodontal and Peri-Implant Diseases and Conditions introduced a new framework that moves beyond the simple diagnosis of “chronic periodontitis” to a multidimensional system of staging and grading (25). Staging assesses the severity and complexity of the disease, while grading evaluates the biological risk of progression (13). ICD codes do not capture this crucial detail, meaning our chronic periodontitis cohort likely comprised a heterogeneous mix of individuals with mild, stable disease (e.g., Stage I, Grade A) and those with severe, rapidly progressing disease (e.g., Stage IV, Grade C). This non-differential misclassification would likely bias our results towards the null, suggesting that the true association between severe, active periodontitis and DPN may be even stronger than the 1.65 hazard ratio we observed. Third, regarding medication effects, while we adjusted for baseline metformin use, we lacked precise longitudinal data to calculate the cumulative duration of exposure or dosage. Furthermore, serum vitamin B12 levels were not routinely screened in all patients, precluding us from directly assessing vitamin B12 deficiency as a potential mediator between long-term metformin use and neuropathy-like symptoms. However, a sensitivity analysis excluding metformin users yielded consistent results, suggesting that our findings are not driven by metformin-associated confounding.

## 5 Conclusion

This study provides strong, longitudinally-derived evidence that chronic periodontitis is a significant and independent risk factor for the development of diabetic peripheral neuropathy among patients with T2DM. This finding moves beyond mere association to establish a temporal link, implicating the systemic inflammatory burden from periodontal disease as a key contributor to the pathogenesis of diabetic microvascular complications. From a clinical perspective, these results advocate for a more integrated approach to diabetes management, where oral health assessment and periodontal care are considered essential components of routine practice. Screening T2DM patients for periodontal disease and providing timely treatment could represent a novel and cost-effective strategy to mitigate the risk of developing debilitating neuropathic complications. For future research, it is imperative to conduct prospective studies that utilize the detailed 2017 staging and grading criteria for periodontitis to explore a potential dose-response relationship between disease severity and the risk of DPN. Ultimately, randomized controlled trials are needed to determine conclusively whether the treatment of chronic periodontitis can prevent or delay the incidence of diabetic peripheral neuropathy.

## Data Availability

The raw data supporting the conclusions of this article will be made available by the authors, without undue reservation.

## References

[1] GBD 2021 Diabetes Collaborators . Global, regional, and national burden of diabetes from 1990 to 2021, with projections of prevalence to 2050: a systematic analysis for the Global Burden of Disease Study 2021. Lancet. (2023) 402:203–34. doi: 10.1016/S0140-6736(23)01301-6, PMID: 37356446 PMC10364581

[2] Yang Y Zhao B Wang Y Lan H Liu X Hu Y . Diabetic neuropathy: cutting-edge research and future directions. Signal Transduct Target Ther. (2025) 10:132. doi: 10.1038/s41392-025-02175-1, PMID: 40274830 PMC12022100

[3] Zhang Y Lazzarini PA McPhail SM van Netten JJ Armstrong DG Pacella RE . Global disability burdens of diabetes-related lower-extremity complications in 1990 and 2016. Diabetes Care. (2020) 43:964–74. doi: 10.2337/dc19-1614, PMID: 32139380

[4] Savelieff MG Elafros MA Viswanathan V Jensen TS Bennett DL Feldman EL . The global and regional burden of diabetic peripheral neuropathy. Nat Rev Neurol. (2025) 21:17–31. doi: 10.1038/s41582-024-01041-y, PMID: 39639140 PMC13011988

[5] Liu X Xu Y An M Zeng Q . The risk factors for diabetic peripheral neuropathy: A meta-analysis. PloS One. (2019) 14:e0212574. doi: 10.1371/journal.pone.0212574, PMID: 30785930 PMC6382168

[6] Bragg S Marrison ST Haley S . Diabetic peripheral neuropathy: prevention and treatment. Am Fam Physician. (2024) 109:226–32. 38574212

[7] Simpson TC Clarkson JE Worthington HV MacDonald L Weldon JC Needleman I . Treatment of periodontitis for glycaemic control in people with diabetes mellitus. Cochrane Database Syst Rev. (2022) 4:CD004714. doi: 10.1002/14651858.CD004714.pub4, PMID: 35420698 PMC9009294

[8] Kudiyirickal MG Pappachan JM . Periodontitis: An often-neglected complication of diabetes. World J Diabetes. (2024) 15:318–25. doi: 10.4239/wjd.v15.i3.318, PMID: 38591080 PMC10999051

[9] Kocher T König J Borgnakke WS Pink C Meisel P . Periodontal complications of hyperglycemia/diabetes mellitus: Epidemiologic complexity and clinical challenge. Periodontol 2000. (2018) 78:59–97. doi: 10.1111/prd.12235, PMID: 30198134

[10] Purnamasari D Khumaedi AI Soeroso Y Marhamah S . The influence of diabetes and or periodontitis on inflammation and adiponectin level. Diabetes Metab Syndr. (2019) 13:2176–82. doi: 10.1016/j.dsx.2019.05.012, PMID: 31235154

[11] American diabetes association. 2. Diagnosis and classification of diabetes: standards of care in diabetes—2024. Diabetes Care. (2024) 47:S20–44. doi: 10.2337/dc24-S002, PMID: 38078589 PMC10725812

[12] Pop-Busui R Ang L Boulton AJM Feldman EL Marcus RL Mizokami-Stout K . Diagnosis and treatment of painful diabetic peripheral neuropathy. In: Feingold KR Anawalt B Blackman MR , editors. Endotext. American: MDText.com, Inc (2022). 35544662

[13] Papapanou PN Sanz M Buduneli N Dietrich T Feres M Fine DH . Periodontitis: Consensus report of workgroup 2 of the 2017 World Workshop on the Classification of Periodontal and Peri-Implant Diseases and Conditions. J Periodontol. (2018) 89:S173–82. doi: 10.1002/JPER.17-0721, PMID: 29926951

[14] Sun J Wang Y Zhang X Zhu S He H . Prevalence of peripheral neuropathy in patients with diabetes: A systematic review and meta-analysis. Prim Care Diabetes. (2020) 14:435–44. doi: 10.1016/j.pcd.2019.12.005, PMID: 31917119

[15] American Diabetes Association . 12. Retinopathy, neuropathy, and foot care: standards of care in diabetes—2024. Diabetes Care. (2024) 47:S231–43. doi: 10.2337/dc24-S012, PMID: 38078577 PMC10725803

[16] Mirea AA ?tefan AG Maria M Clenciu D Mitrea A Efrem IC . The associations of dental and periodontal lesions with the microvascular complications in patients with type 2 diabetes mellitus: A case-control study. Life (Basel). (2024) 14:1585. doi: 10.3390/life14121585, PMID: 39768293 PMC11678667

[17] Mesia R Gholami F Huang H Clare-Salzler M Aukhil I Wallet SM . Systemic inflammatory responses in patients with type 2 diabetes with chronic periodontitis. BMJ Open Diabetes Res Care. (2016) 4:e000260. doi: 10.1136/bmjdrc-2016-000260, PMID: 27651910 PMC5020743

[18] Acharya AB Thakur S Muddapur MV Kulkarni RD . Systemic cytokines in type 2 diabetes mellitus and chronic periodontitis. Curr Diabetes Rev. (2018) 14:182–8. doi: 10.2174/1573399812666161220144011, PMID: 28000545

[19] Preshaw PM Taylor JJ Jaedicke KM De Jager M Bikker JW Selten W . Treatment of periodontitis reduces systemic inflammation in type 2 diabetes. J Clin Periodontol. (2020) 47:737–46. doi: 10.1111/jcpe.13274, PMID: 32106333

[20] Akash MSH Rehman K Liaqat A . Tumor necrosis factor-alpha: role in development of insulin resistance and pathogenesis of type 2 diabetes mellitus. J Cell Biochem. (2018) 119:105–10. doi: 10.1002/jcb.26174, PMID: 28569437

[21] Zhang L Xu F Hou L . IL-6 and diabetic kidney disease. Front Immunol. (2024) 15:1465625. doi: 10.3389/fimmu.2024.1465625, PMID: 39749325 PMC11693507

[22] Reda E Hassaneen S El-Abhar HS . Novel trajectories of bromocriptine antidiabetic action: leptin-IL-6/JAK2/p-STAT3/SOCS3, p-IR/p-AKT/GLUT4, PPAR-γ/adiponectin, Nrf2/PARP-1, and GLP-1. Front Pharmacol. (2018) 9:771. doi: 10.3389/fphar.2018.00771, PMID: 30072896 PMC6058031

[23] Deng R Chen W Zhang Z Zhang J Wang Y Sun B . Association between visceral obesity index and diabetes: A systematic review and meta-analysis. J Clin Endocrinol Metab. (2024) 109:2692–707. doi: 10.1210/clinem/dgae303, PMID: 38709677 PMC11403314

[24] Du Y Xiao H Luo R Li G Ren Y . The role of HbA1c in the bidirectional relationship between periodontitis and diabetes and related interventions: a narrative review. Front Nutr. (2025) 12:1606223. doi: 10.3389/fnut.2025.1606223, PMID: 40599550 PMC12209262

[25] Caton JG Armitage G Berglundh T Chapple ILC Jepsen S Kornman KS . A new classification scheme for periodontal and peri-implant diseases and conditions - Introduction and key changes from the 1999 classification. J Clin Periodontol. (2018) 45 Suppl 20:S1–8. doi: 10.1111/jcpe.12935, PMID: 29926489

